# Assessment of heavy metal (Cu, Ni, Fe, Co, Mn, Cr, Zn) pollution in effluent dominated rivulet water and their effect on glycogen metabolism and histology of *Mastacembelus armatus*

**DOI:** 10.1186/2193-1801-2-390

**Published:** 2013-08-20

**Authors:** Mehjbeen Javed, Nazura Usmani

**Affiliations:** Aquatic Toxicology Research Laboratory, Department of Zoology, Aligarh Muslim University, Aligarh, 202002 India

**Keywords:** Heavy metals, Bioaccumulation, Glycogen, Histopathology, Liver, *Mastacembelus armatus*

## Abstract

The present study was conducted to examine the contamination of rivulet situated at Kasimpur, Aligarh (27.218° N; 79.378° E). It receives the wastewater of Harduaganj Thermal Power Plant (HTPS) containing fly ash and heavy metals. Among the heavy metals estimated in the rivulet water, Fe (8.71 mgL^-1^) was present in the highest concentration followed by Cu (0.86 mgL^-1^), Zn (0.30 mgL^-1^) Mn (0.21 mgL^-1^), Ni (0.12 mgL^-1^), Co (0.11 mgL^-1^) and Cr (0.10 mgL^-1^). The values for the heavy metals such as Fe, Ni and Mn were beyond the limits set by UNEPGEMS. Bioaccumulation of these heavy metals was detected in tissues such as gills, liver, kidney, muscle and integument of the fish *Mastacembelus armatus*. Accumulation of Fe (213.29 – 2601.49 mgkg^-1^.dw) was highest in all the organs. Liver was the most influenced organ and integument had the least metal load. The accumulation of Fe, Zn, Cu and Mn, observed in the tissues were above the values recommended by FAO/WHO. Biochemical estimation related to blood glucose, liver and muscle glycogen conducted showed significant (p < 0.01) elevation in blood glucose content over control (17.73%), whereas liver glycogen dropped significantly (p < 0.01) over control (−89.83%), and similarly muscle glycogen also decreased significantly (p < 0.05) over control (−71.95%), suggesting enhanced glycolytic capacity to fuel hepatic metabolism. Histopathological alterations were also observed in selected organs (gills, liver and kidney) of *Mastacembelus armatus*.

## Introduction

**Figure 1:**
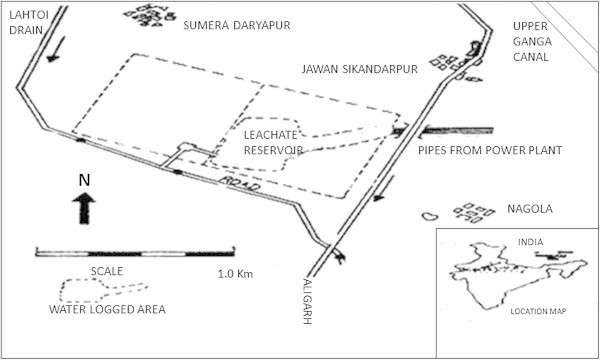
**Map showing the location of Harduaganj Reservoir (27.218° N and 79.378° E) which receives the effluents from the Power Plant via pipes.**

**Figure 2:**
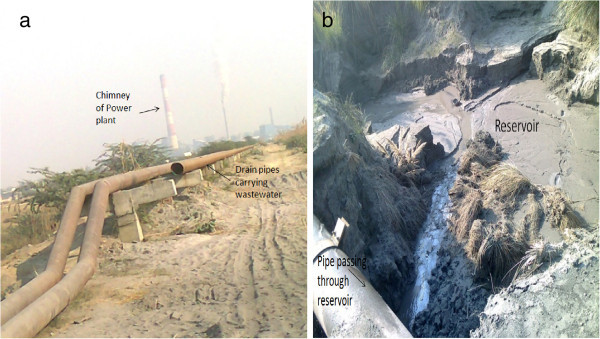
**a) Harduaganj Thermal Power Plant draining wastewater into the reservoir b) Present Scenario: Degraded condition of reservoir.**

**Figure 3:**
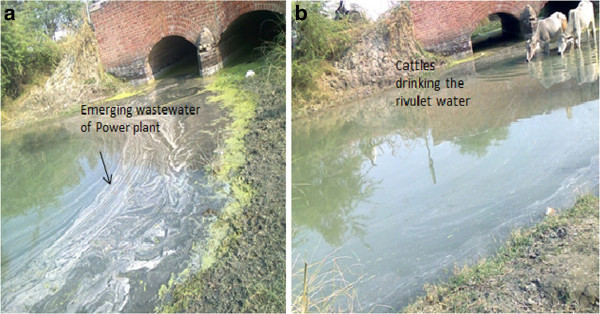
**a) Show wastewater emerging from Power Plant polluting the rivulet b) Rivulet water used for different Purposes.**

Sewage and industrial disposal has greatly increased the addition of heavy metals in the aquatic ecosystems. In Aligarh as well there are very few studies to focus on this aspect. It influences the productivity and health status of water bodies as abnormal changes in physicochemical conditions and other quality parameters have their impact on diversity. Harduaganj Reservoir (27.218° N and 79.378° E) at Kasimpur, (during late 1990’s) Aligarh was quite productive and healthy (Figure 1). Water filled area being 13.5 ha. Now this reservoir is damaged completely because of the discharge of effluents from Harduaganj Thermal Power Station. This has changed the condition of reservoir drastically (Figure 2). We are being deprived of the good resource of water and fish food. It appears more of a waste land. Besides the damage of aquatic ecosystem the fly ash from the power plant has also destroys the terrestrial ecosystem. The area was previously occupied by the lush green crops of mustard, wheat and rice. With time the greenery has been replaced by waste land having ash deposited over it. This all happens due to unsafe disposal of ash from the Power station damaging the soil making it unfit for agriculture. The pipelines from the Power Plant are now used to discharge their wastewaters in one of the adjacent rivulet flowing through the area (Figure 3). Several species of fishes are thriving in such water bodies hence their conservation is essential. The Thermal Power Plant effluent contains gases, fly ash and traces of heavy metals which can endanger the inhabitants. The rivulet water has also been rendered unsafe for fishes as well as for domestic consumption, irrigation, and other needs particularly consumption by cattle and other domestic animals. This attracted a lot of attention as another aquatic ecosystem may get damaged and have the same fate as the reservoir. Therefore the construction of power plants in the areas near crop fields and water bodies should not be prevalent. This will check its impact on both agriculture and aquaculture especially from the capture fisheries point of view and small water bodies usually meet the need of local population.

The aim of this study was to assess the abnormal presence of heavy metals in rivulet water located near Harduaganj Reservoir, their bioaccumulation in different tissues of fish, depletion/increase in carbohydrate levels in blood, muscle and liver and histopathology in organs of the fish. For this purpose, the bioaccumulation of Cu, Ni, Fe, Co, Mn, Cr and Zn were investigated in the fish tissues like gills, liver, kidney, muscle and integument. The histological analysis of gills, kidney and liver was performed in order to confirm the structural damage. Blood glucose, liver and muscle glycogen were also determined as the stress indicators of the fish health. *Mastacembelus armatus* being more prevalent and relished, also of its hardy and tolerant nature and adaptability to various conditions and habitats, was chosen as the model for study.

There is no data on the effect of heavy metals on *Mastacembelus armatus* in general and bioaccumulation, biochemical aspects and histopathology in particular. Data generated from this study will serve as an important reference in the implementation of management strategies against heavy metal pollution, their accumulation in freshwater ecosystems and conservation of aquatic life and water bodies. This study involve the use of already exposed fish from polluted waters which restricts the misuse and unnecessary killing of healthy fishes and also avoid the ethical issues regarding the use of animals in the experiments.

## Materials and methods

### Sample collection

Water was collected in a pre-cleaned and acidified glass bottles and preserved by acidifying with 6 N HNO_3_ (pH maintained to about 2.0) to estimate the presence of heavy metals in the rivulet.

On spot fixation of water was carried out to measure the dissolved oxygen (D.O). Total solids (T.S), total dissolved solids (T.D.S) and total suspended solids (T.S.S) were determined using standard techniques (APHA 2005). The temperature and pH were recorded on the spot using thermometer (Deluxe, 6) and pH strips (S.D Fine chemicals, 0–0.1).

Samples of *Mastacembelus armatus* (n=25) were collected with the help of professional local fishermen. Live specimens of *M. armatus* were transferred to water buckets and brought to the laboratory for further analysis. Sampled fishes were immediately killed, measured and then utilized for analysis of bioaccumulation of heavy metals, histopathology and biochemical parameters.

### Estimation of heavy metals in water samples and fish organs/tissues

Heavy metals (Cu, Ni, Fe, Co, Mn, Cr and Zn) were estimated in rivulet water and fish organs/tissues using Atomic Absorption Spectrometer (Perkin Elmer, Analyst A 800) as per the standard protocols of APHA (2005).

### Biochemical analysis

#### Blood glucose estimation

Fresh fishes were used for blood collection through cardiac puncture and the blood was placed into the sodium fluoride tubes. Blood samples taken were centrifuged at 3500 rpm for 10 min to obtain serum. The glucose levels in the serum were analyzed using the reagent Eco-Pak Glucose (Accurex Biomedical Pvt. Ltd, India). The glucose levels in samples were measured spectrophotometrically (UV–VIS Systronics, 118) against blank at 505 nm.

#### Liver and muscle glycogen estimation

Fresh fishes were dissected to remove the liver and muscles to estimate the glycogen. The glycogen levels in liver and muscle were measured by Anthrone reagent according to the protocols of Carroll et al. (1956).

### Tissue preparation for histological examination

Fresh fishes were dissected to remove the organs (gills, kidney and liver) which were then fixed in bouin’s fluid. Tissues were processed according to the protocols of Humason (1979). All sections were examined and photographed using Nikon Eclipse 80*i* microscope.

### Metal pollution index

The metal Pollution index (MPI) is used to compare the total metals accumulation level in various tissues of the fish when they are beyond five in number. The values were calculated using the equation:

Where, *C*f*n* is the contents for the metal *n* in the sample (Usero et al. 1997)

### Bioaccumulation factor

The bioaccumulation of the heavy metals (HM) in fish tissues were quantified using bioaccumulation factor (BAF). BAF is the ratio of the concentration of a specific heavy metal in the tissue of the organism, to the concentration that heavy metal in the water.

### Statistical analysis

All values are given as Mean ± SD. Statistical differences among the means of heavy metal accumulation in fish tissues were calculated using ANOVA and Duncan’s Multiple Range Test (Duncan 1955). Blood glucose and liver and muscle glycogen estimations were statistically analyzed using student’s t-test (2 tailed) with the help of SPSS 17.

## Results

The average length of fishes measured was 14.20 ± 1.5 cm and average weight was 35 ± 0.42 g.

### Physicochemical parameters and heavy metals in rivulet water samples

Physicochemical characteristics of rivulet water (Temperature = 27.60°C; pH = 6.9; Dissolved oxygen = 6.9 mgL^-1^; Total solids = 652 mgL^-1^; Total dissolved solids = 407 mgL^-1^; Total suspended solids = 245 mgL^-1^) were comparable with ideal water quality. However the heavy metal content in rivulet water were in the order Fe (8.71 mgL^-1^) > Cu (0.86 mgL^-1^) > Zn (0.3 mgL^-1^) > Mn (0.21 mgL^-1^) > Ni (0.12 mgL^-1^) > Co (0.11 mgL^-1^) > Cr (0.10 mgL^-1^) (Table 1) where it was found that Fe, Ni, Mn and Cr exceeded the recommended values set by UNEPGEMS (2006) but Cu and Zn were within the tolerable range.

**Table 1:** **Heavy metal content in rivulet water compared with Quality Guidelines and Standards by International Organization or country**

Heavy metals↓	WHO (guidelines)	USA (standards)	^*^Present study
Cu	2	1.3	0.86±0.01
Ni	0.02	-	0.12±0.02
Fe	-	0.3	8.71±2.88
Co	-	-	0.11±0.02
Mn	0.5	0.05	0.21±.10
Cr	0.05	0.1	0.1±0.02
Zn	3	5	0.3±0.03

All values are in mgL^-1^.

*Values of heavy metal content in the present study are given as Mean±SD, (n=4×3), samples collected from 4 different zones of rivulet and were analyzed in triplicates.

Adapted for Water Quality for Ecosystem and Human Health, 2006 (prepared and published by the United Nations Environment Programme.

Global Environment Monitoring System (GEMS)/ Water Programme).

Blank cells indicate that no, citable information was available.

### Heavy metals in fish tissue samples

Table 2 (superscripts) shows the accumulation of heavy metals in organs of *Mastacembelus armatus*, and subscript shows the accumulation of a heavy metal in various organs of the fish.

**Table 2:** **Accumulation of heavy metals in the organs of** ***Mastacembelus armatus***

Heavy metals	Gills	Liver	Kidney	Muscle	Integument
Cu	^**c**^199.88_**b**_±0.20	^**d**^271.67_**a**_±1.15	^**c**^175.89_**c**_±0.19	^**d**^41.36_**d**_±0.54	^**d**^36.27_**e**_±0.14
Ni	^**c**^200.00_**b**_±1.73	^**c**^449.96_**a**_±0.06	^**d**^149.33_**c**_±0.50	^**c**^58.98_**d**_±0.09	^**c**^45.062_**e**_±0.02
Fe	^**a**^799.66_**b**_±0.41	^**a**^2601.49_**a**_±0.50	^**a**^649.76_**c**_±0.68	^**a**^213.29_**e**_±0.31	^**a**^313.36_**d**_±0.31
Co	ND	^**f**^25.66_**a**_±0.57	ND	^**e**^9.06_**b**_±0.06	^**f**^9.06_**b**_±0.05
Mn	^**d**^25.36_**b**_±0.62	^**e**^49.96_**a**_±0.05	ND	^**e**^9.03_**d**_±0.06	^**e**^13.62_**c**_±0.00
Zn	^**b**^549.33_**b**_±0.57	^**b**^1741.95_**a**_±0.06	^**b**^351.28_**c**_±0.48	^**b**^186.19_**d**_±0.18	^**b**^168.11_**e**_±0.10

Values are Mean±S.D, (n=15), ND= not detected.

Duncans Multiple Range test was used to test the significance among the means.

Means with similar letters (a, b, c, d,e, f) in a column and row are statistically similar at p < 0.01.

*Superscripts indicate accumulation of different heavy metals (mgKg^-1^.dry weight) in particular organs.

**Subscripts indicate accumulation of a heavy metal (mgKg^-1^.dry weight) in various organs.

#### Fe

The concentration of Fe in different organs ranged from 213.29 to 2601.49 mg kg^-1^.dw and it was highest (2601.49 mg kg^-1^.dw) in liver and least (213.29 mg kg^-1^.dw) was recorded in muscles. The order of accumulation was liver > gills > kidney > integument > muscle.

#### Zn

The Zn concentration fluctuates between 168.11 to 1741.95 mg kg^-1^.dw and its pattern of accumulation was liver > gills > kidney > muscle > integument.

#### Ni

Similarly, Ni concentration was highest (449.96 mgkg^-1^.dw) in liver and least (45.06 mgkg^-1^.dw) in integument and the sequence of their presence in organs/tissues were liver > gills > kidney > muscle > integument.

#### Cu

Cu ranged from 36.27 to 271.67 mg kg^-1^.dw in different organs/tissues. Its accumulation was highest in liver and least in integument. The pattern of accumulation was liver > gills > kidney > muscle > integument.

#### Mn

Mn concentration was highest (49.96 mgKg^-1^.dw) in liver and least (9.03 mgKg^-1^.dw) in muscle. The pattern of accumulation was in the order liver > gills > integument > muscle. Mn was not detected in the kidney of *Mastacembelus armatus*.

#### Co

Co concentration was maximum (25.66 mg kg^-1^.dw) in liver followed by muscle (9.06 mg kg^-1^.dw) and integument (9.06 mg kg^-1^.dw). Values obtained for Co in muscle and integument was statistically insignificant. It was not detected in gills and kidney.

Fe accumulation was highest amongst all organs.

All the values recorded for heavy metals accumulation in different organs/tissues were statistically significant (p > 0.01) except for Ni and Cu in gills and Co and Mn in muscles.

According to the metal pollution index calculated for the sampled organs/tissues (Table 3) it was observed that liver (298.68) of the fish most influenced and had highest metal load followed by kidney (278.119), gills (213.63) and integument (46.86). Muscles (44.64) was least influenced by heavy metals. This clearly indicates that each tissue have different capacity of accumulation.

**Table 3:** **Metal Pollution index value of total metal accumulation in organs/tissues of** ***Mastacembelus armatus***

Organs/tissues	Metal Pollution Index (MPI)
Liver	298.68
Kidney	278.19
Gills	213.63
Integument	46.86
Muscle	44.64

Since muscle and integument are edible part and therefore their quality needs to be monitored prior to consumption. The accumulation of heavy metals needed to be compared with recommended levels (Table 4). It was observed that except for Ni the concentrations of Cu, Fe, Mn and Zn are beyond the safe limits.

**Table 4:** **Comparative account of heavy metal concentrations in edible part with standard guidelines**

Heavy metals	^*^Average concentration	Recommended levels (ppm)
Cu	38.815	30 (FAO/WHO 1983)
Ni	52.04	70–80 (USFDA 1993b)
Fe	263.325	100 (FAO/WHO 1989)
Co	9.06	-
Mn	11.325	1.0 (FAO/WHO 1989)
Zn	177.15	100 (FAO/WHO 1989)

*At times along with fish muscle, integument is also consumed therefore average of both is taken.

Blank cells indicate that no information was available.

Table 5 revealed the calculated bioaccumulation factor for different heavy metals in fish tissues. It was found that the concentration of the heavy metals in different tissues of the fish were several folds higher than their concentrations in water. Liver showed high bioaccumulation factor (BAF) for all the heavy metals. In gills the highest BAF was of Zn and in kidney it was Ni. Similarly in muscle the highest BAF was recorded for Zn and least for Fe. In integument highest and least values of BAF were calculated for Zn and Fe respectively.

**Table 5:** **Bioaccumulation factors (BAF)** ^*^ **of heavy metals in the different tissues of** ***Mastacembelus armatus***

Heavy metals↓	Gills	Liver	Kidney	Muscle	Integument
Cu	232.41	315.89	204.52	48.09	42.17
Ni	1666.66	3749.66	1244.41	491.50	375.51
Fe	91.80	298.67	74.59	24.48	35.97
Co	ND	233.27	ND	82.36	82.36
Mn	120.76	237.90	ND	43.00	64.85
Zn	1831.10	5806.50	1170.93	620.63	560.36

.

### Biochemical analysis

It is evident from Table 6 that blood glucose showed significant (p < 0.01) elevation (+17.73%) over control. Whereas liver glycogen content depleted (−89.83%) significantly (p < 0.01) when compared to control. Similarly, muscle glycogen decreased (−71.95%) significantly (p < 0.05) over control.

**Table 6:** **Impact of Heavy metal concentrations on glucose and glycogen content of** ***Mastacembelus armatus***

Tissues	Control	Exposed	Percent change over control
	Glucose/Glycogen content	Glucose/Glycogen content	
Blood (glucose)	1.14±0.01^**^	21.36±0.049^**^	+17.73%
Liver (glycogen)	2.36±0.01^**^	0.24±0.111^**^	−89.83%
Muscle (glycogen)	0.82±0.01^*^	0.23± 0.032^*^	−71.95%

Values are Mean±S.D (n=8); Blood glucose is given in mg%; Glycogen values are given in mg/g; Student’s t- test was used to test the significance **Significant at p < 0.01 and *Significant at p < 0.05; +/− indicates increase or decrease over control.

### Histopathological observations

**Figure 4:**
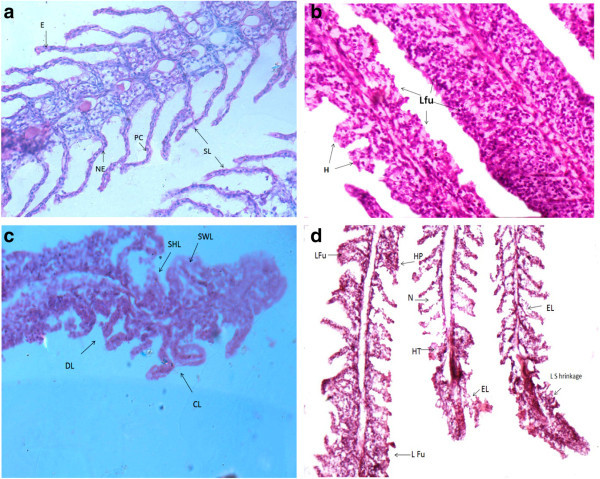
**a) Gills of control** ***Mastacembelus armatus*** **show normal structure of lamellae, erythrocyte (E), nucleus of epithelial cell (NE), pillar cell (PC), secondary lamellae (SL) 400X; Figure b, c and d) Gills of exposed** ***Mastacembelus armatus*** **showing b) complete lamellar fusion (Lfu) coupled with hyperplasia (H) 200X; c) Shrinked lamella (SHL), swelling of lamella (SWL), deformed lamella (DL), curved lamella (CL) 400X; d) Lamellar fusion (Lfu), hyperplasia (HP), necrosis (N), Hypertrophy (HT), epithelial lifting (EL), Lamellae shrinkage (L Shrinkage), Stained with Hematoxylin and Eosin.**

**Figure 5:**
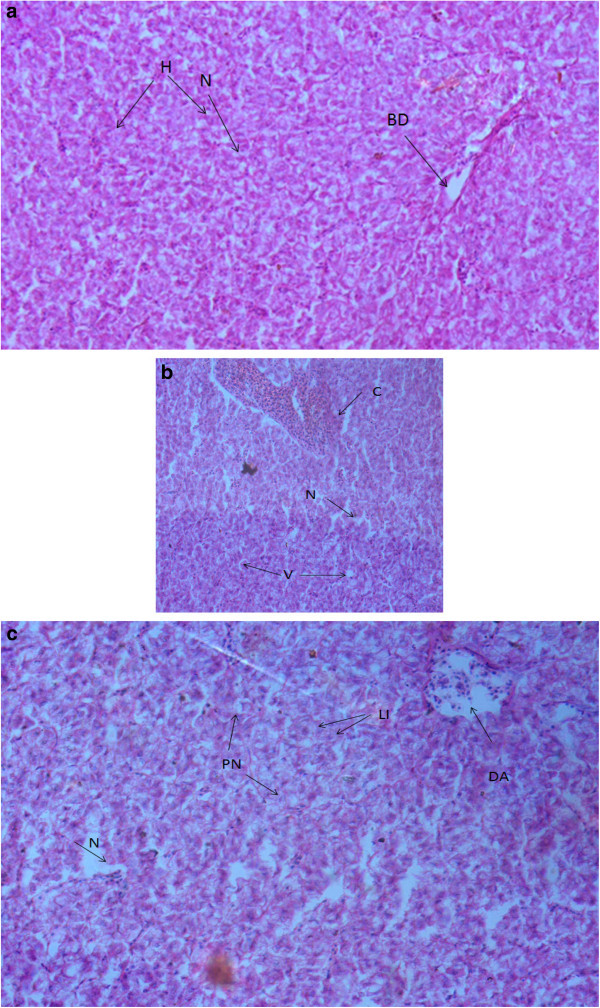
**a) Show control liver of** ***Mastacembelus armatus*** **having hepatocytes (H) with normal nucleus (N), bile duct is also present 400X; Figure b and c) Show liver of exposed** ***Mastacembelus armatus*** **; b) Congestion of blood vessel (C), necrosis (N) and vacuolization (V) of hepatocytes 200X; c) Pyknotic nuclei (PN), infiltration of leucocytes (LI), degeneration of arteriole (DA) and necrosis of parenchyma (N) 400X. Stained with Hematoxylin and Eosin.**

**Figure 6:**
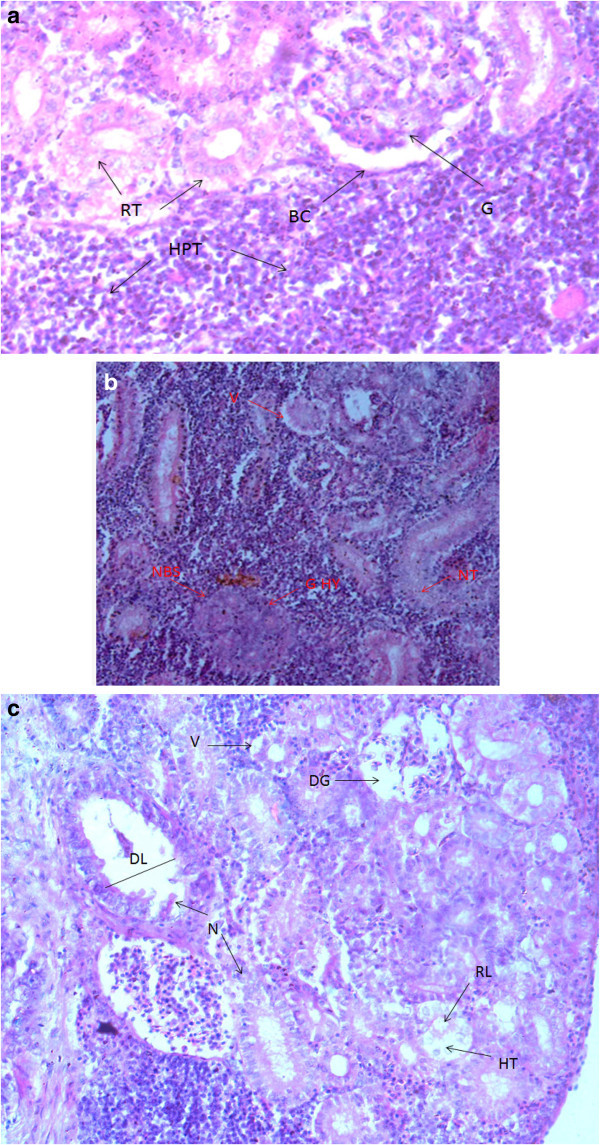
**a) Show control kidney of** ***Mastacembelus armatus*** **having normal structure glomeruli (G) with bowman's capsule (BC), renal tubules (RT) and hematopoeitic tissues (HPT) 400X; Figure b and c) Show kidney of exposed** ***Mastacembelus armatus*** **b) Vacuolization of tubules (V), hyperplasia of glomeruli (GHY) and have no bowman's space (NBS), necrosis of renal tubule (NT) 200X; c) Lumen of tubule dilated (DL), necrosis of hematopoeitic tissue and tubule (N), hypertrophy of tubule (HT) due to which lumen is reduced (RL) 400X; Stained with hematoxylin and eosin.**

Histopathological examinations confirmed the deformities in gills, kidney and liver of the fish and results are shown in Figure 4, 5 and 6. Gill of control fish Figure 4 (a) showed normal structure while exposed fish sections Figure (b,c,d) showed severe lamellar fusion, hyperplasia, hypertrophy and epithelial lifting, swelling and deformed lamella, in some parts sloughing off and curving of lamellae was also observed. Figure 5 (a) Showed liver of control *Mastacembelus armatus* which had network of hepatocytes and bile duct. While Figure 5 (b and c) revealed the histological damage that occurred in liver, necrosis of parenchyma, vacuolation, congestion of blood vessel, pyknosis and infiltration of leucocytes were the major observations. Figure 6 (a) Normal kidney of fish and Figure 6 (b and c) describes the dilation and vacuolation of kidney tubules, decreased lumen of tubule due to hypertrophy, glomeruli with no bowman's space, degeneration of glomeruli and necrosis of hematopoietic tissue.

## Discussion

The water quality of rivulet was suitable for sustenance of fishes as determined by physicochemical characteristics however among the heavy metals examined some were beyond the maximum permissible limits. The heavy metal content estimated in water were present in the order Fe > Cu > Zn >Mn> Ni > Co > Cr where it was found that Fe, Ni, Mn and Cr content exceeded the recommended guidelines set by UNEPGEMS (2006) due to which the water becomes unfit for the inhabitant fishes. There are very few studies which reported presence of heavy metals in waters of Aligarh as well. Javed and Usmani (2012a) reported that water of sewage fed aquaculture pond at Panethi, Aligarh which is used as a source of commercial fish food also contain heavy metals in the order Fe >Mn> Zn > Co > Ni > Cu = Cr. In another study the sugar mill effluent dominated river at village Satha, Aligarh also reported to contain heavy metals in the order Ni > Cr > Cu > Co (Javed and Usmani 2013b). But the metal content in these waters are lower than the present study. Similarly many water bodies which lie in vicinity of population have been polluted by effluents released by industries, factories, Power stations, domestic waste etc. which besides disturbing the quality of water also degrade the protein source in the form of fish food and limits their use (Baki et al. 2011; Abdul Qadir and Riffat Naseem 2011; Javed and Usmani [Bibr CR26][Bibr CR27]; Taweel et al. 2012; Emere and Dibal 2013; Fatima and Usmani 2013). In the present study possible reason for high levels of heavy metals in rivulet water could be attributed to the fact that this Power Station may not have efficient wastewater treatment plant. Although these metals are essential in traces but their excess becomes toxic and gets accumulated in organs/tissues of fish *Mastacembelus armatus*.

### Bioaccumulation of heavy metals in *Mastacembelus armatus*

#### Fe

In the present study it was examined that Fe content was high in water and the same was observed in organs of *Mastacembelus armatus*. It had highest accumulation in liver and least was recorded in muscle. Studies reported for *Clarias gariepinus* (Osman Alaa and Werner 2009), *Tinca tinca* (Selda Tekin et al. 2005), *Labeo rohita* (Javed and Usmani 2011) also revealed the maximum accumulation of Fe in liver and least in muscle. The high Fe concentrations observed in the liver tissue of *M. armatus* could be due to iron-containing enzymes and the extensive vascular system of the liver, as the haemoglobin in the blood binds approximately three quarters of the Fe in the body (Voynar 1960). As compared with the present study the values reported for Fe content in different tissues of *Channa punctatus* inhabiting sewage fed pond, Aligarh was higher and highest accumulation occurred in gills and least in integument (Javed and Usmani 2012a). Accumulation of Fe in muscle of *M. armatus* examined was beyond the permissible limits (100 ppm) set by FAO/WHO (1989).

Fe was highest accumulated metal among Cu, Ni, Co, Mn, Cr, Zn in all the organs/tissues of fish including the present study which may be due to the unique feature of iron metabolism, that there was no route for complete excretion for Fe. Consequently, little iron is lost. High doses of iron are frequently associated with gastrointestinal effects, especially constipation, but also with nausea, diarrhoea and vomiting. Chronic Fe exposure promotes gastric and esophageal ulceration in humans and animals.

#### Zn

Fe accumulation was followed by Zn in organs/tissues of *Mastacembelus armatus*. In the present study it was observed that Zn accumulation was highest in liver and least in integument. Highest accumulation of Zn in liver has also been reported in *Clarias gariepinus* (Coetzee et al. 2002), *Channa punctatus* (Murugan et al. 2008) and *Labeo rohita* (Javed and Usmani 2011) and least in integument of *Channa punctatus* (Javed and Usmani 2012a). The reason for high accumulation of Zn in liver could be due to metallothionein (MT). These are low molecular weight proteins and their production increases due to elevated levels of heavy metals which bind to the metal in order to detoxify them, in doing so they concentrate and regulate metals in liver. In the present study it has also been observed that Zn content was lower in muscle than liver which could be due to the transference of Zn from muscle to other fish organs, and this deloading ability of fish has been reported to be advantageous to fish consumers (Madhusudan et al. 2003; Murugan et al. 2008). Zn accumulation was beyond the permissible limits (100 ppm) set by FAO/WHO (1989) in muscle of *M. armatus*. Although Zn is an essential element as it is carefully regulated by physiological mechanisms in most organisms (Eisler 1988), it is also regarded as a potential hazard that can endanger both animal and human health. Agency for Toxic Substances and Disease Registry (ATSDR 2005), suggests that ingesting high levels of zinc for several months may cause anemia, damage the pancreas, and decrease levels of high-density lipoprotein (HDL) cholesterol.

#### Ni

Ni occupied the third position as far as bioaccumulation was concerned. Highest accumulation was observed in liver and least in integument. In other studies as well the liver reported to accumulate high concentrations of Ni in fishes such as *Labeo umbratus* (Coetzee et al. 2002), *Oreochromis niloticus* (Taweel et al. 2012), *Cirrhinus mrigala* (Javed 2012) and least in integument of *Clarias gariepinus*, *Channa punctatus* (Javed and Usmani 2012a). Accumulation of Ni in muscle of *M. armatus* was below the permissible limits (70–80) set by USFDA (1993b). Ni content has also been reported to be beyond the permissible limits in water of Yamuna River at Okhla barrage as well in fishes *Channa striatus* and *Heteropneustes fossilis* (Fatima and Usmani 2013). Ni exhibits similar chemical behavior as Fe and Co. Concentrations of Ni in water is likely to be of health concern in environments where pH is less than 4.5. As is the case with other essential elements Ni is also toxic to fish when present in high enough concentrations (Pickering 1974). An uptake of too large quantities of nickel results in respiratory failure, Birth defects, Asthma and chronic bronchitis, Heart disorders etc. This can also cause various kinds of cancer on different sites within the bodies of animals.

#### Cu

Highest accumulation of Cu also occurred in liver and least in integument. Other workers also reported the highest copper content in liver of fishes *Labeo umbratus* (Coetzee et al. 2002), *Oncorhynchus mykiss*, *Cyprinus carpio* (Boeck et al. 2004), *Wallago attu* (Yousafzai et al. 2010). The Cu content observed in the present study in muscle is similar to accumulation observed in *Clarias gariepinus* (Anim et al. 2010) and *Wallago attu* (Yousafzai et al. 2010). According to Stokes (1979), fish muscles have poor accumulative properties, even in systems containing high copper levels. The accumulation of Cu in muscle was beyond the permissible limits (30 ppm) set by FAO (1983). But in another study from sugar mill effluent dominated water body at Satha, Aligarh, the Cu content observed in edible part of *Channa punctatus* was below the permissible limits (Javed and Usmani 2013b). Copper is essential for good health. However, exposure to higher doses can be harmful. Cu toxicity in natural water arising from pollutants may cause severe damage in gills and necrotic changes in the liver and kidneys. According to ATSDR (2005), long term exposure to Cu, higher than normal levels can cause nausea, vomiting, stomach cramps, or diarrhea.

#### Mn

The highest accumulation of Mn was observed in liver and least in muscle. However other workers investigated highest accumulation of Mn in gills and least in muscle of fishes such as *Oreochromis mossambicus* (Robinson and Oldewage 1997), *Tinca tinca* (Selda Tekin et al. 2005), *Clarias gariepinus* (Osman Alaa and Werner 2009), *Cyprinus carpio* (Jabeen and Chaudhry 2010), *Clarias anguillaris* (Nwajei et al. 2012). Accumulation observed in muscle in present study corroborates to *Oreochromis mossambicus* (Robinson and Oldewage 1997) and *Clarias gariepinus* (Coetzee et al. 2002). The content of Mn examined in muscle of *M. armatus* was several folds higher than the maximum permissible limits (1.0 ppm) of Mn set by FAO/WHO (1989). High levels of Mn can cause lung, liver and vascular disturbances, declines in blood pressure, failure in development of animal foetuses and brain damage. Finally, laboratory tests with test animals have shown that severe manganese poisoning should even be able to cause tumor development in animals.

#### Co

Co was the least accumulated metal in tissues of *Mastacembelus armatus*. The highest accumulation occurred in liver while muscle and integument were least influenced. Other workers reported high accumulation of Co in kidney of fishes *Channa punctatus* and *Labeo rohita* but comparable levels in muscle and integument (Javed and Usmani 2011). Smith and Carson (1981) reported that Co is mostly accumulated (about 75%) in the viscera and integument of the fish and the statement agrees well with the present study. No permissible guidelines/limits have yet been established for Co. Generally, cobalt compounds that dissolve easily in water are more harmful than those that are hard to dissolve in water. Once cobalt enters the body, it is distributed into all tissues, but mainly into the liver, kidney, and bones (ATSDR 2004).

#### Cr

In the present study it was not detected in any of the fish organs/tissues though it was reported beyond permissible limits in rivulet water. However high concentrations had been reported in the water of sugar mill effluent dominated water body at Satha, Aligarh as well as in prevalent fish *Channa punctatus* (Javed and Usmani 2013b). Similarly Fatima and Usmani (2013) also reported Cr content in both water and fishes (*Channa striatus* and *Heteropneustes fossilis*) of Yamuna at Okhla barrage, which was beyond the permissible limits.

In this study, liver was observed to be the most influenced organ. Lower concentrations in muscle and integument possibly indicate that the fish integument is an important excretory organ for these metals and also serve as a protective barrier between outer environment and muscle. But still the accumulated amount is of concern.

### Bioaccumulation factor (BAF)

Bioaccumulation factor was used to quantify the metal concentrations in fish organs/tissues relative to the concentrations in rivulet water. In the present study the BAF values was high. It was calculated highest for liver (233.27- 5806.5) and least for integument (35.97- 560.36) and following the order, liver > gills > kidney > muscle > integument. This showed that liver had high metal load than other organs.

### Effects of heavy metals on glycogen metabolism

The Thermal Power Plant effluents brought about a number of significant changes in the carbohydrate metabolism in *Mastacembelus armatus*. There was significant elevation in blood glucose over control (+17.73%) while glycogen in liver (−89.83%) and muscle (−71.95%) decline significantly. This hyperglycemic (increase in blood glucose) condition were also detected in fishes, *Heteropneustes fossilis* and *Saccobranchus fossilis*, exposed to Ni and Cr (Nath and Kumar 1988; Radhakrishaniah et al. 1992), *Labeo rohita* and *Clarias gariepinus* subjected to Cu (Van Vuren et al. 1994; James et al. 1996), *Oreochromis aurues* exposed to mixture of Cu and Pb salts. During the present study increase in blood glucose content as a result of heavy metals present in power plant effluent could be attributed to the enhanced glycogenolysis, resulting in formation of glucose to meet the energy demand during stress.

In the present study liver and muscle glycogen dropped significantly under the stress of heavy metals. Srivastava and Srivastava (2008) reported that glycogen consistently decreased from 8.18 to 5.3 mg g^-1^in *Channa punctatus* when exposed to sublethal concentrations of ZnSO_4_. Similarly, other studies also subscribes to the above view in fishes such as *Channa punctatus* subjected to distillery effluent (Maruthi and Subba Rao 2000), *Mystus cavasius* to electroplating industrial effluent (Palanisamy et al. 2011). In this study it has been noticed that liver has highest Metal Pollution Index therefore it is assumed that high accumulation levels of heavy metals in liver impaired the activity of enzymes which contribute to glycogen synthesis, leading to decrease in glycogen content.

### Histopathology

The present study revealed that Thermal Power Plant effluents induced histopathological alterations in gills, liver and kidney of *Mastacembelus armatus*. Under present investigation, it has been observed that gills of fish exhibited several histological alterations like complete fusion of lamellae coupled with hyperplasia, hypertrophy and epithelial lifting, necrotic and shrinked/curved lamellae. Similar results have been reported in fishes such as *Labeo rohita* exposed to Cr (VI) (Sesha Srinivas V and Rao BM 1998), *Tilapia mossambica* to Cu, Ni, Cr (Ravanaiah and Narasimha Murthy CV 2010), *Cyprinus carpio* to Cr (Parvathi et al. 2011), *Clarias gariepinus* to sewage and domestic wastewater containing Cu, Fe, Pb, Cd and Zn (Authman et al. 2013). The alterations observed in gill of *M. armatus* prove that it was under chronic exposure and these changes could be interpreted as defense responses of the fish, as these alterations increase the distance across which the dissolved heavy metals must diffuse to reach the bloodstream. Prolonged exposure to heavy metals can lead to degeneration of the epithelium. It can therefore be argued that gill epithelium was the principal entry point of contamination which on exposure to heavy metals multiplied causing hyperplasia.

In the present study the liver of fish also exhibited the severe histopathological lesions like deshaped hepatocytes, vacuolization, necrosis of parenchyma, pyknosis and infiltration of leucocytes. Similar results have been reported in liver of different fishes, *Oreochromis mossambicus* exposed to cadmium and zinc (Van Dyk et al. 2007), *Clarias gariepinus* exposed to fuel oil for 14 days (Gabriel 2007), *Channa punctatus* exposed to hexavalent chromium (Mishra and Mohanty 2008). *Clarias batrachus* to ZnSO_4_ (Prasanna Subhas Joshi 2011), *Cyprinus carpio* to lethal concentrations of Cr (Parvathi et al. 2011), *Tilapia zilli* to Al (Hadi and Alwan 2012), *Clarias gariepinus* to sewage/ domestic wastewater containing Cu, Fe, Pb, Cd, Mn and Zn (Authman et al. 2013). It has been noticed that only toxicant exposed liver show vacuolation and pyknosis (Karyomegaly). Degeneration of liver tissue and necrosis could be due to the infiltration of leucocytes and according to Hughes et al. (1979) necrosis is the direct toxic effect of the pollutant. Further, the histopathological picture of the liver of *M. armatus* corroborates with the biochemical changes accounting for the functional disruption on the activity of the organ due to cellular damage.

Similarly, kidney of *M. armatus* exhibits dilation and vacuolation of kidney tubules, hypertrophy of renal tubules, degeneration of glomeruli and necrosis of hematopoietic tissue. These degenerative changes in kidney were also reported in various fishes such as *Anabas testudineus* exposed to unused lignite mine (Supap Saenphet 2009), *Clarias batrachus* to ZnSO_4_ (Prasanna Subhash Joshi 2011), *Cyprinus carpio* to lethal concentrations of Cr (Parvathi et al. 2011). The main function of kidney is washing/filtration of body fluids and to maintain the homeostasis. Severity of lesions observed in the present study showed that uriniferous tubules and hematopoietic tissue was badly damaged which could impair the renal function and as a consequence heavy metals get accumulated in various organs or muscle of the fish.

## Conclusion

The effluent from the power plant containing heavy metals influenced the water quality of the rivulet under study. Concentrations of heavy metals assessed Fe, Ni, Mn and Cr were found to exceed the permissible limits set for water quality for ecosystem and human health. These constructions not only influence quality of water, but they directly influence flora and fauna where in this case it was observed that a major protein source in the form of fish *Mastacembelus armatus* was influenced which is the major diet of local population. These heavy metals also influence fish physiology. Hence, some scientific method of detoxification is essential to improve the health of these economic fish in the stressed environmental conditions. Since virtually all metals investigated were found in higher concentration, so government should intact laws which will ensure that industries make use of standard waste treatment plants for the treatment of their wastes before they are being discharged into water bodies. Some monitoring programs should also be launched from time to time in order to prevent the misuse of valuable water resources, to check their water quality status, and to restore them for the welfare of society and to protect the natural environment. Since these fishes share the local market therefore they must be screened by food agencies before they reached the humans in order to avoid the epidemics as occurred in Japan (1956) due to consumption of heavy metal contaminated fish and fishery products. Further studies are suggested particularly on the reproductive aspects of the fish in order to check its reproductive health/potential which will help to conserve the species.
